# Extremely Sensitive Dependence of SnO_x_ Film Properties on Sputtering Power

**DOI:** 10.1038/srep36183

**Published:** 2016-11-08

**Authors:** Yunpeng Li, Qian Xin, Lulu Du, Yunxiu Qu, He Li, Xi Kong, Qingpu Wang, Aimin Song

**Affiliations:** 1 Center of Nanoelectronics and School of Microelectronics, Shandong University, Jinan 250100, China; 2 Suzhou Institute of Shandong University, Suzhou, 215123, China; 3 School of Electrical and Electronic Engineering, University of Manchester, Manchester M13 9PL, United Kingdom

## Abstract

An extremely sensitive dependence of the electronic properties of SnO_x_ film on sputtering deposition power is discovered experimentally. The carrier transport sharply switches from n-type to p-type when the sputtering power increases by less than 2%. The best n-type carrier transport behavior is observed in thin-film transistors (TFTs) produced at a sputtering power just below a critical value (120 W). In contrast, at just above the critical sputtering power, the p-type behavior is found to be the best with the TFTs showing the highest on/off ratio of 1.79 × 10^4^ and the best subthreshold swing among all the sputtering powers that we have tested. A further increase in the sputtering power by only a few percent results in a drastic drop in on/off ratio by more than one order of magnitude. Scanning electron micrographs, x-ray diffraction spectra, x-ray photoelectron spectroscopy, as well as TFT output and transfer characteristics are analyzed. Our studies suggest that the sputtering power critically affects the stoichiometry of the SnO_x_ film.

Transparent oxide-semiconductor thin-film transistors (TFTs) have already been commercialized to replace amorphous silicon (a-Si) for backplane drivers in flat-panel displays. There is also huge potential in other integrated circuits and high-frequency applications on glass or flexible substrates due to their low-deposition temperature, high field-effect mobility, and transparency in the visible wavelength range^1^,^2^,^3^. Most oxide semiconductors are n-type, such as amorphous indium gallium zinc oxide (a-IGZO)^4^ and ZnO^5^. Only a very limited number of oxide semiconductors exhibit p-type conduction. P-channel TFTs are necessary in order to fabricate transparent CMOS circuits. Recently, some metal oxides have proven to be promising p-type oxide semiconductors such as Cu_2_O^6^ and SnO^7^,^8^. SnO exhibits excellent p-type conductivity due to the effective overlap of Sn 5*s* orbitals at the valance band maximum^7^. So far, CMOS inverters using n-type semiconductors (such as SnO_2_, ZnO) and p-type SnO TFTs have been fabricated^9^,^10^. CMOS-like inverters based on bipolar SnO TFTs have also been demonstrated^11^,^12^,^13^. In general, however, p-type metal-oxide semiconductors are much less studied than their n-type counterparts. The reported TFT performance, particularly the on/off ratio, *I*_on_/*I*_off_, is yet to be satisfactory for most practical applications.

To date, different deposited methods were adopted to fabricate SnO films. The first device-quality SnO film was deposited using pulsed laser deposition (PLD) with a single-phase SnO ceramic target^7^, and the first SnO-based ambipolar oxide TFT was also fabricated through PLD^11^. However, it is not easy to deposit large-area thin films using PLD in commercial manufacturing. Electron-beam evaporation was also used for SnO films with a high-purity SnO_2_ ceramic source, based on the reaction 2SnO_2_⇋SnO + O_2_^8^,^12^,^14^,^15^,^16^,^17^. However, the as-deposited SnO film required high-temperature post-annealing at more than 300 °C in Ar ambient or 600 °C in vacuum^17^. Electron-beam evaporation is also not ideal for commercial large-area film deposition. Sputtering, on the other hand, is a very well established thin-film deposition technique in industry. SnO TFTs fabricated with DC magnetron sputtering^18^,^19^ exhibited a mobility, *μ*, more than 6 cm^2^V^−1^s^−1^ and an on/off ratio more than 10^4^. Radio-frequency (RF) magnetron sputtering was also used to deposit SnO recently. Polycrystalline SnO ceramic plate^10^, Sn metallic target^13^,^20^,^21^, Sn/SnO_2_ mixed target^22^, and even ceramic SnO_2_ target^23^ have been used as the source of RF magnetron sputtering. Post-annealing was still required but the temperature was generally not higher than 300 °C^10^,^19^. SnO is thermodynamically unstable at temperatures higher than 270 °C because of the escalated disproportionation reaction (4SnO → Sn_3_O_4_ + Sn → 2SnO_2_ + 2Sn)^22^,^23^,^24^. In addition, a low process temperature is necessary for flexible electronics. To the best of our knowledge, the only reported flexible SnO TFT was fabricated through DC magnetron sputtering^19^. Effects of different oxygen partial pressures during sputtering and post-annealing in different gas/vacuum environments have also been studied^19^,^21^,^25^,^26^.

To date, hole mobilities above 10 cm^2^V^−1^s^−1^ have been achieved in SnO^27^, fairly comparable to the typical carrier mobilities of n-type metal oxide semiconductors such as ZnO and IGZO. However, the on/off ratios of most of SnO TFTs reported to date^7^,^13^,^19^,^28^, are only around 10^3^, many orders of magnitude lower than their n-type counterparts where an on/off ratio higher than 10^9^ has been demonstrated^29^. By using a double-gated structure, the on/off ratio of SnO TFT was recently improved^18^ to 10^5^. The TFT off state current determines the minimum device power consumption and is important to transparent oxide integrated circuits for future applications. So far, very limited studies have focused on the possible causes of low on/off ratios of SnO TFTs. Ogo *et al*. suggested that it could be due to trap states deeper than 0.2 eV above the valence band edge, because the Fermi level could not be raised further at large positive gate voltages^28^. In crystalline SnO, the defects are mainly composed of tin vacancies, oxygen vacancies, tin interstitials (Sn_i_)^30^. Tin vacancies contribute to acceptor-like shallow defect states which produce p*-*type conductivity of SnO. The oxygen vacancies may cause shallow defect states near the valence band maximum and conduction band minimum. First-principle calculations suggested that oxygen vacancy concentration in SnO should be orders of magnitude smaller than that of tin vacancies, making them unlikely to quench the p-type conductivity^30^. Experiments have indicated that a large trap density in the mid-gap region can significantly affect the TFT on/off ratio because of the small indirect bandgap of SnO^28^. Such mid-gap trap states may be attributed to Sn_i_ present in the film^30^. Interestingly, a study indicated that the presence of Sn_i_ could enhance the mobility^19^, because they may modify the valence band and contribute more to the delocalized Sn 5*s* and Sn 5*p* orbitals as compared to the localized O 2*p* orbitals^31^. In this study, SnO_x_ films are deposited by radio-frequency magneton sputtering at a range of powers and the fabricated TFTs are annealed at different temperatures. We show that a slight decrease or increase in sputtering power by a few percent can result in an abrupt transition from p-type conduction to n-type conduction as well as a drastic change in the on/off ratio. The study suggests that the main cause of a high off-current (*I*_off_) in p-type SnO TFTs is the presence of Sn_i_ in the film. Enhancements of both the hole mobility and *I*_off_ caused by Sn_i_ are observed in our experiment.

## Results and Discussion

### Dependence of the electronic properties of SnO_x_ TFTs on sputtering power

SnO_x_ films were sputtered using a metallic Sn target in Ar/O_2_ gas at different sputtering powers from 100 to 150 W corresponding to power densities from 2.63 to 3.29 W/cm^2^. A heavily doped p-type silicon wafer with a 300-nm thermally grown SiO_2_ as the gate dielectric was used for the SnO_x_ TFTs. The as-deposited TFTs were annealed in air at different temperatures of 150, 175, 200, 225, 250, 275 and 300 °C.

**Figure 1:**
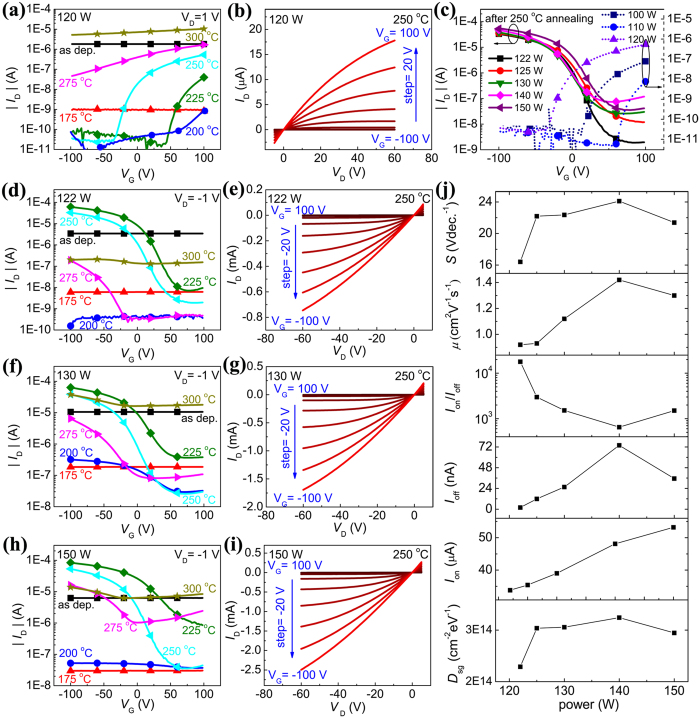
Performance of SnO_x_ TFTs. (**a**) Transfer curves of SnO_x_ TFT sputtered at 120 W after annealing at different temperatures. (**b**) Output curves of SnO_x_ TFT sputtered at 120 W after annealing at optimal temperature of 250 °C. (**c**) Comparison of the transfer curves of SnO_x_ TFTs sputtered at 100, 110, 120, 122, 125, 130, 140, 150 W after annealing at 250 °C. (**d**) Transfer curves of SnO_x_ TFT sputtered at 122 W after annealing at different temperatures. (**e**) Output curves of SnO_x_ TFT sputtered at 122 W after annealing at 250 °C. (**f**) Transfer curves of SnO_x_ TFT sputtered at 130 W after annealing at different temperatures. (**g**) Output curves of SnO_x_ TFT sputtered at 130 W after annealing at 250 °C. (**h**) Transfer curves of SnO_x_ TFT sputtered at 150 W after annealing at different temperatures. (**i**) Output curves of SnO_x_ TFT sputtered at 150 W after annealing at 250 °C. (**j**) Important parameters (subthreshold voltage swing *S*, mobility *μ*, on/off ratio *I*_on_/*I*_off_, on current *I*_on_, off current *I*_off_, and subgap trap density of states *D*_sg_) of p-type TFTs sputtered at different powers after annealing at 250 °C.

**Figure 2:**
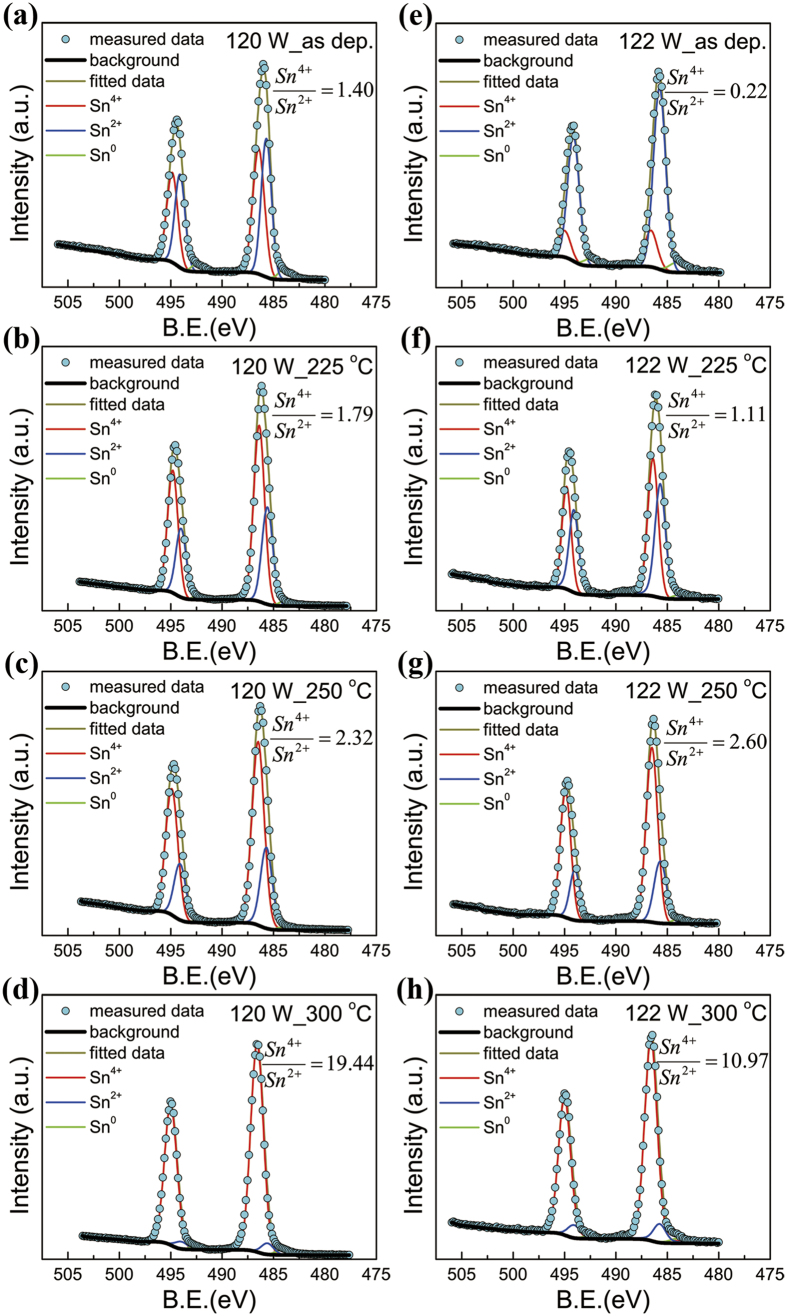
XPS results of the 27 nm-thick n-type SnO_x_ thin film sputtered at 120 W (**a–d**) and p-type SnO_x_ thin film sputtered at 122 W (**e–h**) without and with annealing at different temperatures in the air in the Sn 3*d*_5/2_ and 3*d*_3/2_ core level regions: (**a,e**) as-deposited, (**b,f**) annealed at 225 °C, (**c,g**) annealed at 250 °C, and (**d,h**) annealed at 300 °C. (B.E. is the binding energy from Fermi level. Sn^4+^/Sn^2+^ values correspond to the ratio of the area of two Sn^4+^ peaks and two Sn^2+^ peaks.)

**Figure 3:**
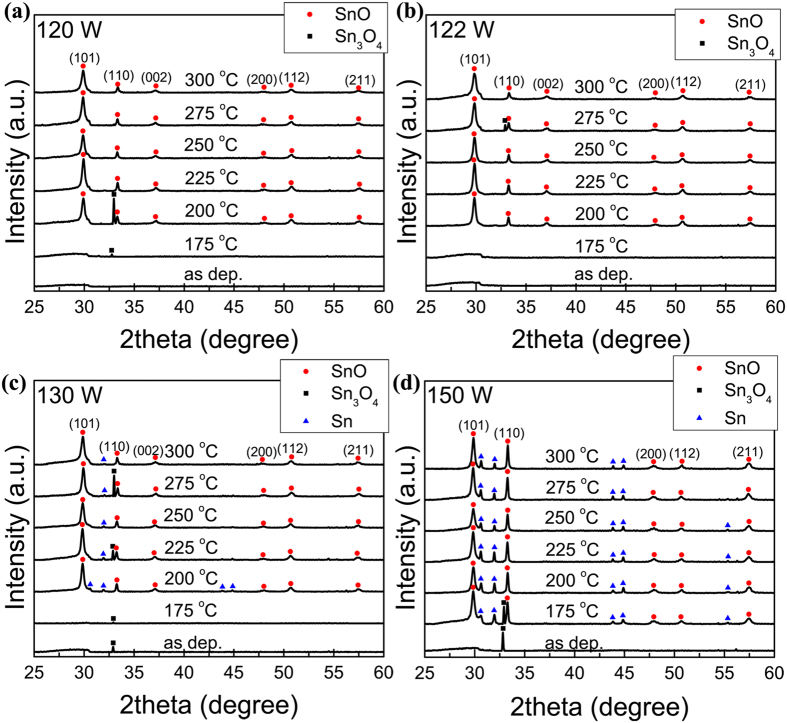
XRD patterns of SnO_x_ films. XRD patterns of 1-μm-thick as-deposited and annealed (175, 200, 225, 250 °C) SnO_x_ films sputtered at (**a**)120, (**b**)122, (**c**)130, and (**d**) 150 W.

Figure 1(a) shows the transfer curves of the annealed SnO_x_ TFT that were sputtered at 120 W. The as-deposited SnO_x_ layer was very conductive and the source-drain current *I*_D_ could hardly be tuned by gate voltage. Annealing at 175 °C decreased *I*_D_ by three orders of magnitude but the gate still showed little tuneability. After annealing at and above 200 °C, pronounced field effect could be observed. Both the transfer and output characteristics in Fig. 1(a,b) exhibit n-type behavior indicating that the film is predominantly SnO_2_. The X-ray photoelectron spectroscopy (XPS) results in the Sn 3*d*_5/2_ and 3*d*_3/2_ core level regions, as shown in Fig. 2(a–d), confirm the existence of SnO_2_ in the film. The peaks originated from Sn^4+^, Sn^2+^, and Sn^0^ were centered at binding energies of 494.8 and 486.4 eV, 494.0 and 485.6 eV, and 492.4 and 484.0 eV, respectively^15^,^21^. Gaussian-dominated fitting (with Lorentz ratio <20%) was applied to deconvolute the contributions of Sn^4+^, Sn^2+^, and Sn^0^. Previous work found that the carrier transport switched from p-type to n-type as the result of transformation from SnO to SnO_2_ like structures and finally to SnO_2_^10^. The optimal n-type performance was obtained after annealing at 250 °C with *I*_on_/*I*_off_ = 1.89 × 10^4^ and *μ* = 0.02 cm^2^V^−1^s^−1^. The low mobility of the n-type sample sputtered at 120 W may be because the predominant composition of SnO_2_ was amorphous indicated by the lack of crystalline SnO_2_ in the XRD patterns in Fig. 3(a). Furthermore, the SnO component existed in the film, as shown in the XRD patterns in Fig. 3(a) and XPS spectra in Fig. 2(a–d). SnO elements may act as electron traps and hence reduce the electron mobility of SnO_2_. The dramatic increase in *I*_D_ after annealing at 275 and 300 °C by orders of magnitude is likely a result of the disproportionation reaction^22^,^23^,^24^. The resulting metallic Sn makes the film more conductive and more difficult to be tuned by the gate voltage. Films deposited at different sputtering powers below 120 W all exhibited n-type behavior but with lower on/off ratios as shown in Fig. 1(c).

In contrast, p-type behavior originated in SnO was always obtained when the sputtering power was at or above 122 W. Figure 1(d) shows the transfer curves of the SnO_x_ TFT sputtered at 122 W after annealing at different temperatures. The as-deposited film was also very conductive and could not be tuned by gate voltage. *I*_D_ decreased after further annealing at 175 and 200 °C while the TFTs still did not show any obvious field effect. After annealing at 225 °C, typical p-type characteristics were observed with a hole mobility of 1.40 cm^2^V^−1^s^−1^ and on/off ratio of 9.10 × 10^3^. The highest on/off ratio, 1.79 × 10^4^, was obtained after 250 °C annealing with a hole mobility of 0.92 cm^2^V^−1^s^−1^. Such an on/off ratio is among the highest values reported to date in SnO TFTs with a single gate^18^. The output characteristic is shown in Fig. 1(e). Further annealing at 275 and 300 °C led to performance degradation as shown in Fig. 1(d) because of the disproportionation reaction^22^,^23^,^24^. Figure 1(f) to 1(i) show the transfer and output curves of the SnO_x_ TFTs sputtered at 130 and 150 W. Similar dependences on the annealing temperature was observed but with much lower on/off ratios as shown in Fig. 1(c).

Detailed performance parameters (subthreshold voltage swing *S*, *μ*, *I*_on_/*I*_off_, *I*_on_, *I*_off_, and subgap trap density of states *D*_*sg*_) of the TFTs fabricated at different sputtering powers after annealing at 250 °C are summarized in Fig. 1(j) and Supplementary Table S1. It is observed that in comparison to *I*_on_, *I*_off_ shows a much more sensitive dependence on the sputtering power. Furthermore, *S* value decreases as the sputtering power decreases, indicating a decrease of *D*_sg_ in the channel layer and the interface as *S* = *ln*10(*k*_*B*_*T*/*q*)(1 + *qD*_*sg*_/*C*_*G*_)^29^. Here, *q* is the electron charge, *k*_*B*_ is the Boltzmann constant, *T* is the temperature, and 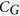 is the gate capacitance. The obtained value of *D*_sg_ for the p-type SnO TFTs was in the order of 10^14^ cm^−2^eV^−1^, making it difficult to raise the Fermi level under positive gate biases, resulting in a high *I*_off_^28^. Furthermore, *I*_off_ and *D*_sg_ are positively correlated as shown in Fig. 1(j), which is consistent with the findings by Ogo *et al*.^28^.

The decreases of *I*_off_, *μ*, and *S* at lower sputtering powers (122, 125, 130, and 140 W) can be attributed to the change in the Sn_i_ concentration^28^,^30^,^31^,^32^. First, the Sn:O ratio decreases as the sputtering power decreases^33^. Sn-rich SnO films are likely to form at high sputtering powers because the sputtered Sn atoms from the target can travel at high speeds and may not be sufficiently oxidized when reaching the substrate. On the other hand, O-rich SnO films are likely formed at low sputtering powers. According to first-principle calculations the formation energy of Sn_i_ is lower in Sn-rich films than in O-rich films^30^. As a result, Sn_i_ forms more easily in Sn-rich films produced at high sputtering powers than in O-rich films at low sputtering powers. Furthermore, Sn_i_ is expected induce a huge density of states in the bandgap^30^, leading to a high *I*_off_^28^. As such, both *S* and *I*_off_ were found to decrease with the diminution of sputtering power. Moreover, Sn_i_ can improve the hole mobility by enhancement of delocalized Sn 5*s* and 5*p* orbitals in the valence band maximum^31^, and hence *μ* was found to decrease as the sputtering power decreased. The dependence of Sn_i_ density in the film on the sputtering power and the slight anomaly at a sputtering power of 150 W will be discussed further with XRD and SEM analysis.

**Figure 4:**
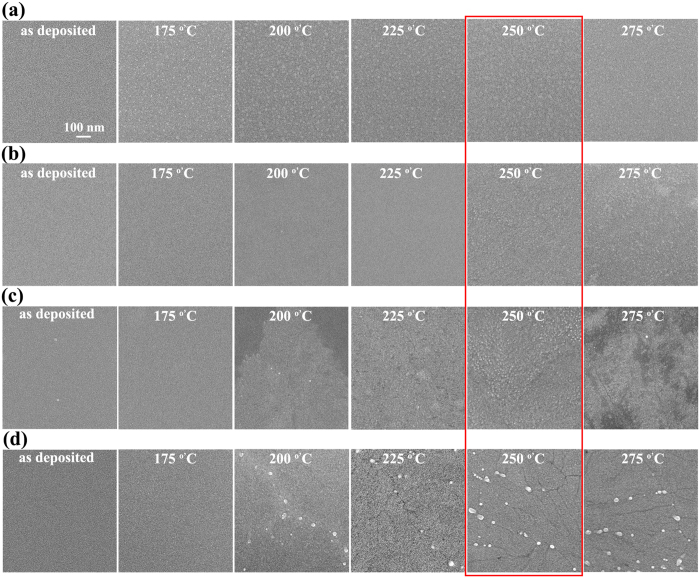
Surface morphologies of SnO_x_ films. SEM images of as-deposited and annealed (175, 200, 225, 250, and 275 °C) SnO_x_ channels of TFTs sputtered at (**a**) 120, (**b**) 122, (**c**) 130, and (**d**) 150 W. The scale is same for all the images, and is shown in (**a**).

Supplementary Table S1 shows the key parameters, *I*_on_, *I*_off_, *I*_on_/*I*_off_, *S*, *μ*, and *D*_sg_, of p-type TFTs with SnO sputtered at different powers after annealing at 225 and 250 °C in air. Films annealed at both temperatures showed very similar dependence on the sputtering power. At the higher annealing temperature, the tin-to-oxygen ratio in the film is expected to be lower due to further oxidation of SnO and excess metallic Sn^21^ in the film. It is therefore reasonable that films annealed at 225 °C contained more Sn_i_ than those annealed at 250 °C, and this is confirmed by XPS analysis as shown in Fig. 2(f,g). The topmost ultrathin layer of the SnO_x_ film (~1 nm) should be the native oxidized SnO_2_ layer^15^, and the photoelectron inelastic mean free path under the Al-*K*_α_ X-ray source (1486.6 eV) is ~2 nm. Hence, Sn^4+^ component has been overestimated in the XPS results, but the increased ratio of Sn^4+^/Sn^2+^ can confirm an increase of the SnO_2_ component and a decrease of the SnO component with the increasing annealing temperature, as shown in Fig. 2 (e–h). Thereby, the atomic tin-to-oxygen ratio (Sn/O) decreases as the annealing temperature increases, and this agrees very well with that reported by Cho *et al*.^21^. As a result, the SnO_x_ film gradually transformed from Sn-rich to O-rich when the film was annealed in the air with increasing temperatures. According to the first-principle calculations, the formation energy of Sn_i_ in Sn-rich films is much lower than that in O-rich films^30^. Thus, the amount of Sn_i_ in the 250 °C sample should be less than that in the 225 °C sample. This is further supported by the formation of Sn clusters, which should partly originate from Sn_i_, in the 250 °C sample as shown in the SEM results (Fig. 4). Sn_i_ defects are expected to enhance the hole mobility, because they modify the valence band maximum by improving the ratio of delocalized Sn 5*s* and 5*p* orbital contributions to localized O 2*p* orbital contribution^19^,^31^. Thereby, the hole mobility decreased as the annealing temperature increased from 225 to 250 °C. In addition, as the annealing temperature increases, the increased amount of n-type SnO_2_ can act as hole traps and thus also decreases the hole mobility. The increase of on/off ratio is attributed to the decrease amount of Sn_i_, when the p-type samples were annealed from 225 to 250 °C. Sn_i_ defects are expected to induce a huge density of states in the bandgap^30^, which makes it difficult to raise the Fermi level under positive gate bias^28^. Thus, lower Sn_i_ concentrations in the p-type samples annealed at 250 °C than those in the samples annealed at 225 °C leads to lower off currents and consequently higher on/off ratios, as shown in Fig. 1(d,f,h) and Supplementary Table S1.

### Dependence of the microstructures of SnO_x_ films on sputtering power

One-μm-thick SnO_x_ films sputtered at various sputtering powers were prepared for XRD analysis. Figure 3(a) shows the XRD patterns of as-deposited and annealed SnO_x_ films sputtered at 120 W. The as-deposited film is obviously amorphous. A single peak, attributed to Sn_3_O_4_^32^,^34^, was observed after annealing at 175 °C. The Sn_3_O_4_ may be intermediate oxidation state when SnO was oxidized to SnO_2_ although pronounced disproportionation reaction was mostly observed at higher temperatures^22^,^23^,^24^. Clear SnO peaks were observed after annealing at 200~300 °C, but SnO_2_ rather than SnO should be the dominant phase in the film because separate TFTs sputtered and annealed under the same conditions all showed n-type conduction. SnO_2_ is amorphous at this temperature and cannot be detected by XRD^35^,^36^, but can be confirmed by XPS as shown in Fig. 2. The Sn_3_O_4_ peak disappeared after annealing at 225 °C, indicating the completion of the disproportionation reaction and oxidation from Sn_3_O_4_ to SnO_2_.

Figure 3(b) shows the the XRD patterns of the as-deposited and annealed SnO_x_ films sputtered at 122 W. SnO peaks were detected in films annealed at 200~300 °C, and a Sn_3_O_4_ peak was observed after annealing at 275 °C. In films sputtered at higher sputtering powers (Fig. 3(c,d)), both Sn and SnO peaks were observed in XRD spectra. These results agree well with previous XPS analysis, i.e., the percentage of metallic Sn in the film increases as sputtering power increases^33^. It is worth noting that the crystallization of films sputtered at 150 W was detected at a lower annealing temperature of 175 °C, indicating that crystallization occurred more easily in the film sputtered at higher powers. This was most likely due to higher kinetic energy of sputtered atoms and hence stronger ability of self-organizing during the film formation. Similar dependence of crystallinity on sputtering power was also observed in ref. 33.

A small Sn_3_O_4_ peak was detected in as-deposited films sputtered at 130 W, and a much stronger Sn_3_O_4_ peak was observed when the sputtering power reached 150 W. This is likely due to sputtered atoms having higher kinetic energies, making it easier to reach the intermediate oxidation stage^32^,^37^. Previous work showed that Sn_3_O_4_ could behave as an n-type semiconductor with dominant electron transport mechanism described by variable range hopping^38^. Further work is needed to study the influence of the Sn_3_O_4_ phase to SnO TFTs.

Figure 3 also indicate a stronger presence of pure Sn phase at higher sputtering powers, particularly 130 and 150 W. Sn atoms are expected to be sputtered at higher rates (as shown in Supplementary Fig. S4) and hence less likely to be thoroughly oxidized before reaching the substrate, resulting in excess Sn. Overall, the XRD spectra indicated that the composition of the film was not pure but with coexistence of Sn, SnO, Sn_3_O_4_ and SnO_2_. However, the electrical properties in Fig. 1 indicate that SnO_2_ is the dominant phase in the films sputtered at 100, 110, and 120 W, and SnO is the dominant phase at 122, 130, 140, and 150 W.

### Dependence of the surface morphologies of SnO_x_ films on sputtering power

Figure 4(a) shows the scanning electron micrograph of as-deposited and annealed SnO_x_ films (27 nm thick for TFTs) sputtered at 120 W. The as-deposited film was quite smooth. Tiny bright spots were observed in the film after annealing at 175 °C, which we speculate to be metallic Sn clusters. Given that the as-deposited film sputtered at 120 W was very conductive, there might be some metallic Sn in the film which could not be detected by XRD. Such tiny bright spots gradually disappeared after further annealing, which might be due to the oxidation process of metallic Sn to amorphous SnO_2_.

Figure 4(b–d) show the surface morphologies of as-deposited and annealed p-type SnO films sputtered at 122 W, 130 W, and 150 W, respectively. The as-deposited films were all quite smooth. Obvious change of the surface morphology occurred after annealing at 250 °C, 200 °C, and 200 °C for films sputtered at 122 W, 130 W, and 150 W, respectively. The change of the surface morphology could be related to the crystallization of SnO and the formation of Sn clusters at the grain boundaries or dislocations. Furthermore, some cracks were observed in annealed film sputtered at 150 W (Fig. 4(d)) which could be related to grain boundaries^25^.

The optimal n-type or p-type TFT performance was obtained after annealing at 250 °C (Fig. 1 and the red box in Fig. 4), where the suspected Sn clusters became larger as sputtering power increased. This is consistent with the presence of more Sn as shown in the XRD spectra in Fig. 2. It can also be observed that such Sn clusters were mostly distributed along the cracks (Fig. 4(d)). In general, excess Sn may exist in the SnO_x_ film in the forms of Sn_i_ in the SnO lattice and Sn clusters, which may and may not be at the grain boundaries, dislocations, *etc*^19^. Sn clusters are not continuous and should not significantly affect the TFT properties. Sn_i_ can affect the off current by forming the mid-gap states as discussed above.

Our experiments showed that TFTs fabricated at a sputtering power of 150 W showed a slightly higher on/off ratio than those TFTs fabricated at 140 W. Sn clusters are expected to form more easily at structural defects, such as the cracks shown in SEM images in Fig. 4(d). As a result, less Sn interstitials are expected to remain after annealing induced clustering, leading to fewer mid-gaps states and a lower off current. Fewer Sn interstitials would contribute less to the Sn 5*s* and Sn 5*p* orbitals compared to the localized O 2*p* orbitals^31^, resulting in a slightly reduced mobility as shown in Fig. 1(j) and Supplementary Table S1.

## Conclusion

We have fabricated n- and p-type SnO_x_ TFTs by precisely controlling the sputtering power. Our experiments revealed that even a tiny increase of the sputtering power above the critical value could sharply switch the film conduction from n- to p-type. The highest on/off ratio of our p-type SnO TFT was 1.79 × 10^4^, which is among the best values reported to date in single-gated TFTs. Our XRD/XPS/SEM analysis suggested that the high on/off ratio could be related to the formation of Sn clusters and the resulting reduction of Sn interstitials and the related mid-gap states, which occurred most strongly at an annealing temperature of 250 °C. The revealed extremely sensitive dependence of SnO film properties on sputtering conditions may provide useful clues to future work in order to further reduce the off current of P-type SnO TFTs which remains a key bottleneck issue in achieving satisfactory oxide-semiconductor CMOS circuits for practical applications.

## Methods

### Film growth and characterization

SnO_x_ layers were deposited by radio frequency magnetron sputtering using a metallic Sn target. The target is 3 inches in diameter with an area of 45.60 cm^2^. The substrate temperature was 100 °C. The working pressure during sputtering process was ~4.8 mTorr with a fixed Ar/O_2_ mixture atmosphere at a ratio of 23/3. The thickness of SnO_x_ films for TFTs and SEM imaging was 27 nm, and the thickness of SnO_x_ films for XRD was 1 μm. The sputtering powers were set at 100, 110, 120, 122, 125, 130, 140, and 150 W. The microstructure of the SnO_x_ films was characterized by X-ray diffraction (XRD, D8 Advance). XPS (ESCALAB 250) measurements with an Al-*K*_α_ X-ray source were carried out to determine the chemical components of the film.

### Device fabrication and characterization

A heavily doped p-type silicon wafer was used both as substrate and gate electrode. 300-nm thermally grown SiO_2_ was employed as the gate dielectric. Bottom gate TFT structures were fabricated using shadow mask. The source and drain electrodes were 50 nm-thick Pd deposited by an electron-beam evaporator. The active channel length and channel width were fixed at 60 and 2000 μm, respectively. Nine devices were fabricated at each of sputtering powers. The devices were annealed in air at 150, 175, 200, 225, 250, 275, and 300 °C step by step, and the annealing time was 1 hour at each temperature. The surface morphology of channel layer was analyzed using a scanning-electron microscope (FEI Nova NanoSEM 450). Device characteristics of the TFTs were measured using a source/measure unit (Agilent B2902A) in dark.

## Additional Information

**How to cite this article**: Li, Y. *et al*. Extremely Sensitive Dependence of SnO_x_ Film Properties on Sputtering Power. *Sci. Rep.* **6**, 36183; doi: 10.1038/srep36183 (2016).

**Publisher’s note:** Springer Nature remains neutral with regard to jurisdictional claims in published maps and institutional affiliations.

## Supplementary Material

Supplementary Information
