# Supplementary material for: Extremely Sensitive Dependence of SnOx Film Properties on Sputtering Power
Source: Sci Rep. 2016 Nov 8;6:36183. doi: 10.1038/srep36183 (PMC5099937; doi:10.1038/srep36183)
Supplement: Supplementary Information [file srep36183-s1.pdf]

Supplementary Materials

Extremely Sensitive Dependence of SnO_x Film Properties on Sputtering Power

Yunpeng Li[1], Qian Xin[1,2*], Lulu Du[1], Yunxiu Qu[1], He Li[1], Xi Kong[1], Qingpu Wang[1], and Aimin Song[1,2,3*]

[1]Center of Nanoelectronics and School of Microelectronics, Shandong University, Jinan 250100, China

[2]Suzhou Institute of Shandong University, Suzhou, 215123, China

[3]School of Electrical and Electronic Engineering, University of Manchester, Manchester M13 9PL, United Kingdom

[*]Corresponding author: Qian Xin
E-mail: Xinq@sdu.edu.cn
[*]Corresponding author: Aimin Song
E-mail: A.Song@manchester.ac.uk

Power (W)	Annealing temperature (°C)	I_{on} (A)	I_{off} (A)	I_{on}/I_{off}	S (Vdec.$^{-1}$)	μ ($cm^2V^{-1}s^{-1}$)	D_{sg} ($cm^{-1}eV^{-1}$)
122	225	6.19E-5	6.80E-9	9103	15.15	1.40	2.08E14
	250	3.38E-5	1.89E-9	17884	16.32	0.92	2.25E14
125	225	5.50E-5	7.28E-8	755	26.18	1.42	3.59E14
	250	3.54E-5	1.19E-8	2975	22.64	0.93	3.04E14
130	225	6.44E-5	3.65E-7	176	31.76	1.56	4.36E14
	250	3.90E-5	2.56E-8	1523	25.08	1.12	3.07E14
140	225	9.16E-5	6.31E-7	145	30.84	2.21	4.23E14
	250	4.81E-5	7.39E-8	651	24.11	1.42	3.31E14
150	225	8.49E-5	8.57E-7	99	49.38	1.76	6.78E14
	250	5.32E-5	3.54E-8	1503	21.39	1.30	2.93E14

Table S1. Electronic parameters of p-type SnO TFTs sputtered at different sputtering powers after annealing at 225 and 250 °C

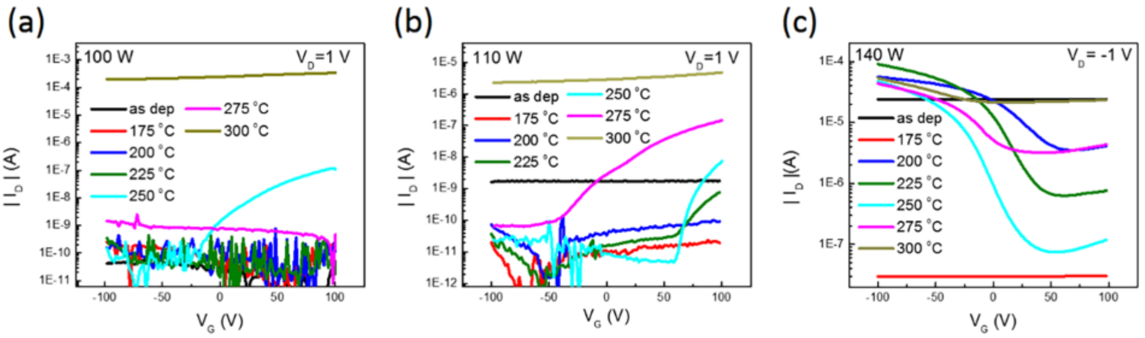

Figure S1. Variation of transfer curves for SnO_x TFTs sputtered at powers of a) 100 W, b) 110 W, c)140 W after annealing at different temperatures.

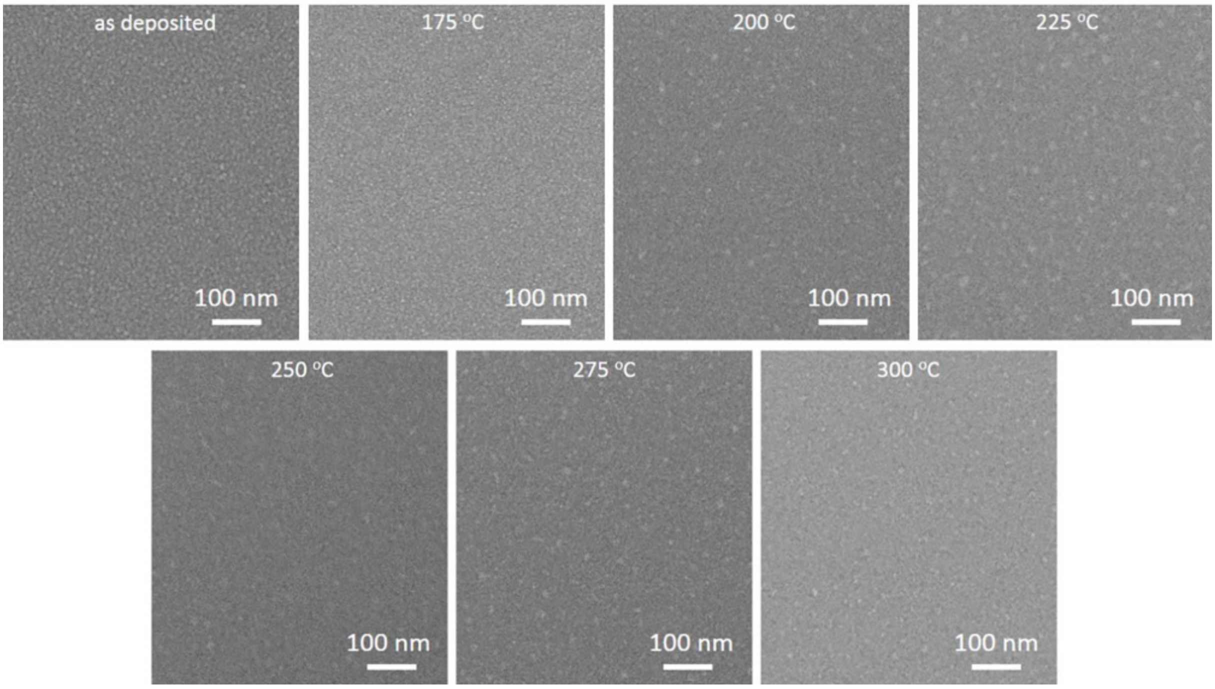

Figure S2. SEM images of as-deposited and annealed (175, 200, 225, 250, 275 and 300 ^{o}C) SnO_x channels of TFTs sputtered at 110 W

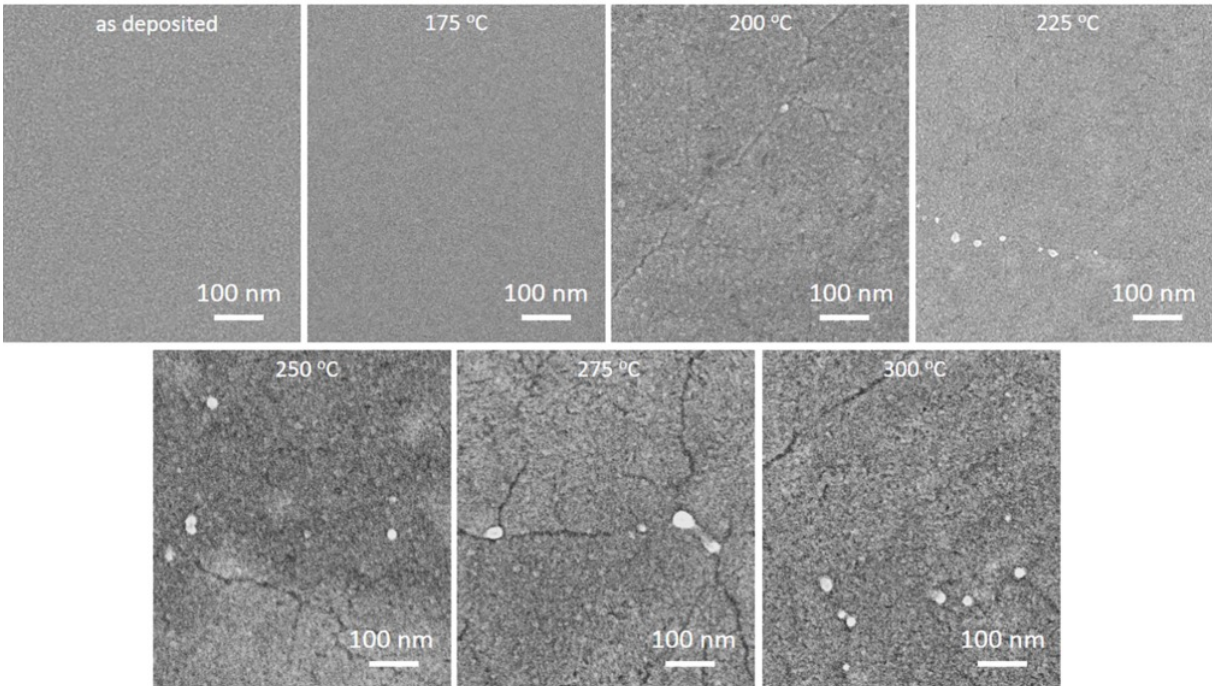

Figure S3. SEM images of as-deposited and annealed (175, 200, 225, 250, 275 and 300 ^{o}C) SnO_x channels of TFTs sputtered at 140 W

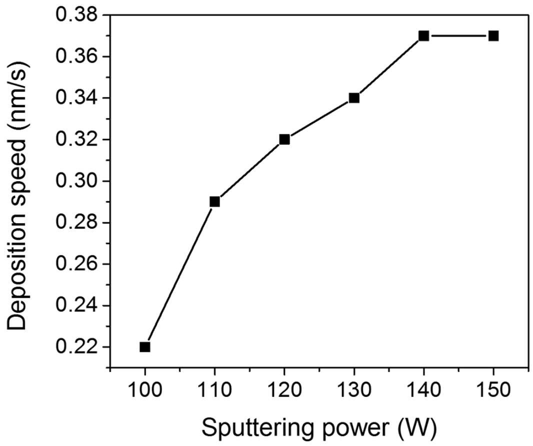

Figure S4. The dependence of the deposition speed on sputtering power

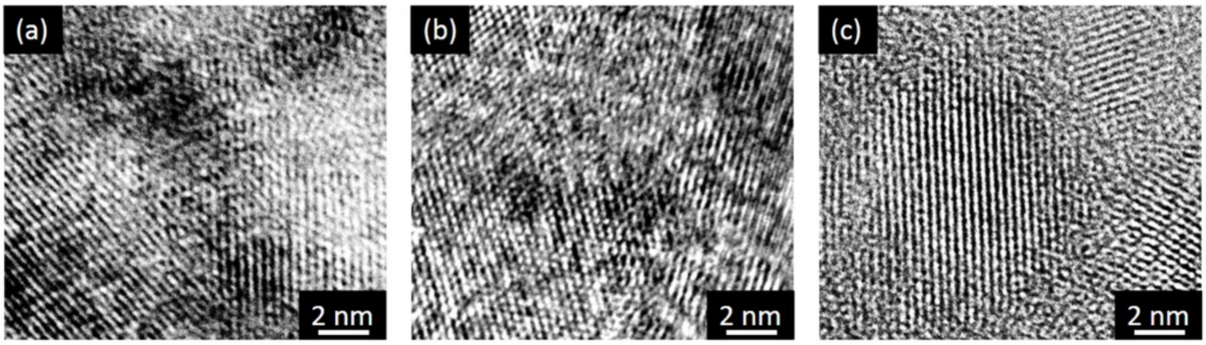

Figure S5. TEM images of SnO thin films sputtered at a) 122 W, b) 130 W, c) 150 W after annealing at 250 °C

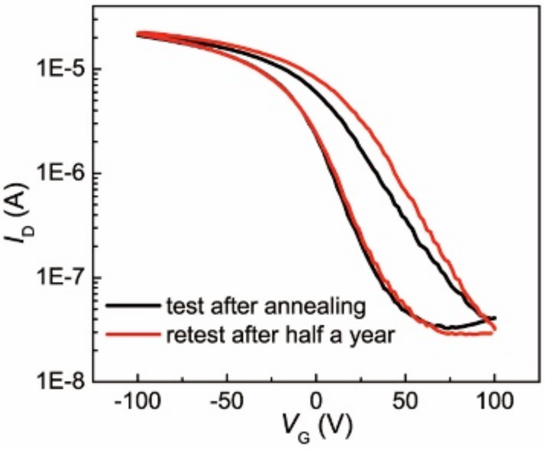

Figure S6. Electrical stability of the TFTs with the sputtering power of 150 W after half a year.
